# Regulatory and Influencing Factors of Digestive Function in Elderly People: Roles of the Gut Microbiota and Nutritional Interventions

**DOI:** 10.14336/AD.2025.0565

**Published:** 2026-06-05

**Authors:** Ka Li, Safia Arbab, Qiujing Du, Jiao Zhou, Yuwen Chen, Yali Tian, Li Qijie, Hanif Ullah, Ben Zhang

**Affiliations:** ^1^West China School of Public Health/West China Fourth Hospital, Sichuan University, Chengdu, Sichuan, China.; ^2^Medicine and Engineering Interdisciplinary Research Laboratory of Nursing & Materials/Nursing Key Laboratory of Sichuan Province, West China Hospital, West China School of Nursing, Sichuan University, Chengdu, Sichuan, China.; ^3^Hainan General Hospital and Hainan Affiliated Hospital, Hainan Medical University, Haikou, China

**Keywords:** Influencing factors, Digestive function, Elderly people, Gut microbiota, Nutrition

## Abstract

Aging is a natural and gradual biological process through which living organisms undergo physical, physiological, and sometimes psychological changes over time. Aging is commonly associated with a decline in gastrointestinal function, leading to various digestive disorders that impact the quality of life of older adults. The gut microbiota is a highly complex ecosystem that plays crucial roles in digestion, metabolic processes, immune functions, and overall health. However, emerging evidence indicates that many elderly individuals maintain relatively stable digestive health, suggesting the influence of modifiable regulatory factors. In this review, we describe the key physiological, microbial, and nutritional factors that regulate and influence digestive function in an aging population. Additionally, we explored the impact of age-associated alterations in the gut microbiota on digestive health challenges in older adults and emphasized the therapeutic potential of targeted nutritional intervention approaches, such as dietary modifications, prebiotics, probiotics, and symbiotic and fecal microbiota transplantation, which have shown promise in rebalancing the gut microbiome and reducing inflammation.

## 1. Introduction

Aging is a complex biological process that involves gradual physiological changes across multiple organ systems, with the gastrointestinal (GI) tract being notably affected by alterations such as reduced digestive secretions, delayed motility, and impaired nutrient absorption, which collectively challenge digestive efficiency in elderly people [1]. Despite these changes, many older adults maintain relatively stable digestive function, indicating that various regulatory and influencing factors, including the gut microbiota and nutrition, genetic factors, lifestyle, diet, and environmental influences, play critical roles in maintaining gastrointestinal health during aging. Genetic factors certainly extend lifespan, and it is becoming increasingly clear that elderly people exhibit distinct biological adaptations that help them maintain health and function well beyond their life expectancy [2]. Interestingly, the GI system plays a central role in nutrient absorption, metabolism, and overall health. It is important to understand what factor prevents the digestive function of elderly people from degrading compared with typical aging-derived reductions. The digestive system experiences several changes as individuals age, including a decrease in gastric motility, reduced enzyme secretion, slower intestinal transportation, and changes in the composition of the gut microbiota. Aging people have slower digestion, lower acid production, and reduced enzyme activity, which can lead to indigestion and constipation [3]. This review aims to examine the regulatory and influencing factors of digestive function in elderly people, with a particular focus on the roles of the gut microbiota and nutritional interventions that support digestive health, promote longevity, and improve overall quality of life in the aging population.

## 2. Aging and Its Impact on Digestive Function

### 2.1 Digestion and absorption

In aging people, the gastrointestinal tract undergoes various structural changes, including mucosal atrophy, decreased gastric acid secretion, and compromised gut wall integrity, which collectively contribute to reduced motility, impaired nutrient absorption, nutrient insufficiency, and an increased risk of digestive disease [3,4]. The reason for this is called anorexia of aging, where older people tend to eat less because they burn less energy due to having a lower metabolism and reduced physical activity [5]. Chewing efficiency decreases with age due to factors such as tooth loss, gum disease, reduced salivary secretion, and loss of taste sensitivity, which collectively contribute to decreased appetite and an elevated risk of malnutrition; although esophageal function is largely maintained, age-related conditions such as impaired lower esophageal sphincter relaxation can lead to dysphagia and delayed gastric emptying [6]. Digestive secretions, such as gastric acids, pancreatic enzymes, and bile acids, generally remain stable with healthy aging [6,7]. Research indicates that pancreatic size starts to decrease after age 60, but this does not necessarily lead to a decline in pancreatic function, especially in healthy individuals [7]. Bile acid secretion does not appear to be significantly impaired by aging. However, aging people are more susceptible to comorbidities such as cholelithiasis and *Helicobacter pylori* infections. Cholelithiasis affects 15% to 25% of individuals over 65 years of age and may interfere with bile acid release [6].

### 2.2 Gastric motility and secretion

Age-related declines in gastric motility and digestive enzyme secretion commonly result in delayed gastric emptying and slower digestion, contributing to symptoms such as indigestion and nutrient malabsorption. However, the ability of some older individuals to manage these changes effectively may be attributed to a combination of favorable genetic factors and sustained healthy lifestyle practices. A recent systematic review highlighted several typical age-associated gastrointestinal diseases, including reduced peristaltic contractility in the distal esophagus and impaired relaxation of the lower esophageal sphincter, both of which are potential contributors to the increased prevalence of gastroesophageal reflux disease (GERD) in the aging population [6]. The prevalence of GERD increases with age, with estimates indicating that approximately 15-40% of individuals over the age of 50 are affected, a trend that may be attributed to age-related alterations in esophageal function as well as other physiological and anatomical changes associated with aging [6,8]. Several studies suggest that aging can slow gastric emptying, but others point to the opposite phenomenon because the meals used in the research vary. Information on elderly people’s transit time in the colon has also provided mixed results, some suggesting that transit is longer [9,10]. However, various age-related factors, including medication use, reduced physical activity, and low dietary fiber intake, have been shown to slow transit time in elderly people [10]. A study reported that a neurodegenerative change in the enteric nervous system may contribute to altered intestinal motility in older people, although the findings have been inconsistent and require further investigation [11,12]. Endocrine disorders common in aging, such as type 2 diabetes and hypothyroidism, have been shown to reduce gastric and intestinal motility. These conditions may partly explain the changes in gastrointestinal motility observed with aging. Gastric acid secretion decreases with age, often due to atrophic gastritis or prolonged medication use, leading to impaired absorption of key nutrients such as B vitamins, iron, and calcium and increased susceptibility to bacterial overgrowth [13].

### 2.3 Morphological and functional changes in the intestine

**Figure 1.**
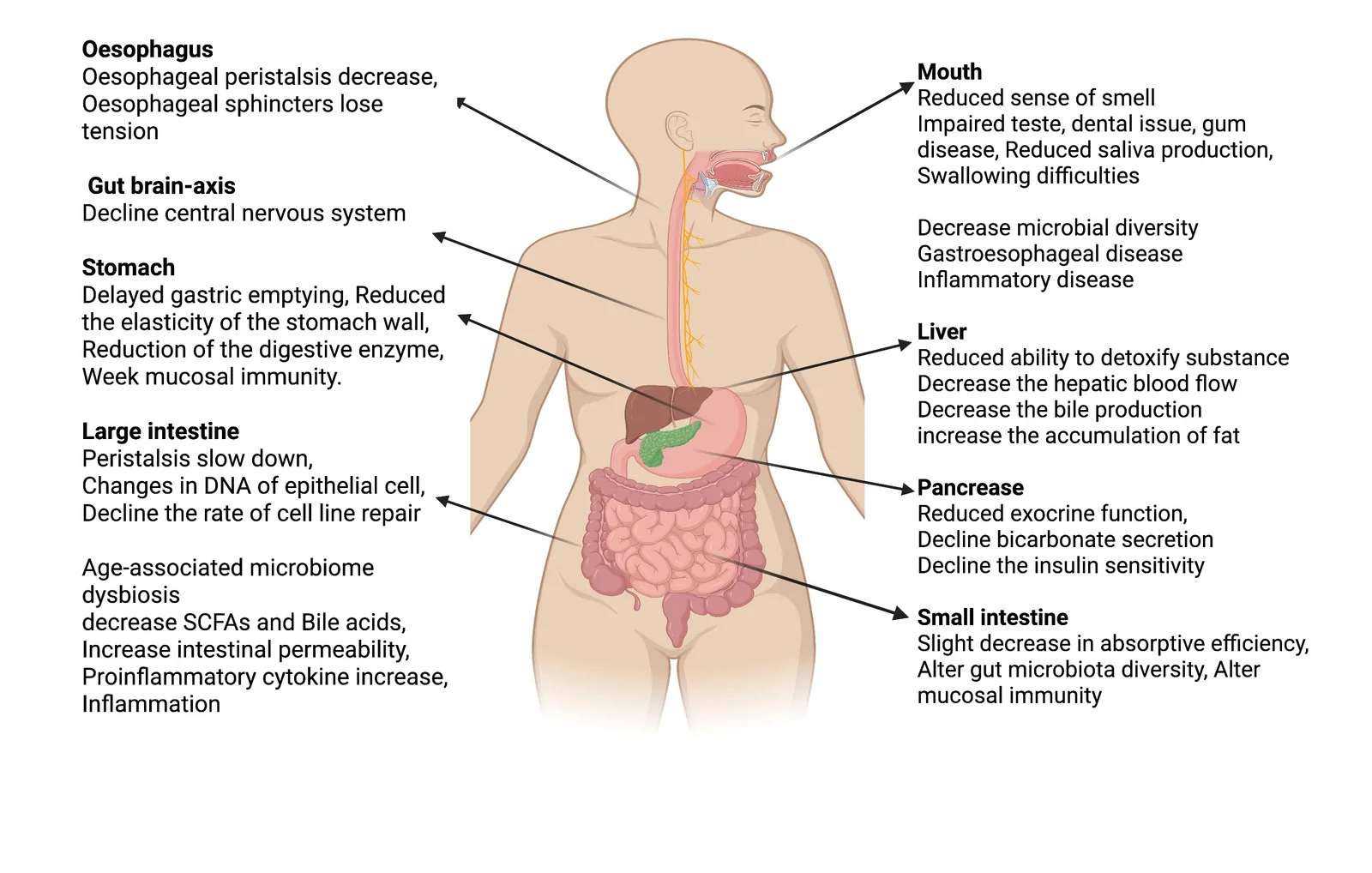
Aging-related changes in the gastrointestinal system.

Aging causes significant morphological and functional changes in the gastrointestinal tract (GIT), impacting digestion and nutrient absorption. However, in aging people, there is villus atrophy, decreased crypt depth, and reduced epithelial regeneration of the intestine. However, it is functionally associated with alterations in the chemical composition of the gut microbiota, nutrient absorption, immune function, and decreased inflammatory and intestinal permeability [6]. Its structure and function change with age, as evidenced by studies in both animal models and humans. In the small intestine, motility and absorption are largely unaffected, although mucosal regeneration may slow. Colonic motility shows mixed findings, with some elderly individuals experiencing constipation or incontinence, which is influenced by neural degeneration or inactivity. Pancreatic exocrine function diminishes with age, as indicated by reduced enzyme output and increased fecal elastase-1 but typically does not cause severe maldigestion. Structurally, the pancreas may exhibit atrophy and fat infiltration. Endocrine function is mostly maintained in nondiabetic patients, although insulin resistance tends to increase, resulting in elevated glucose levels. (Fig. 1) illustrates age-related gastrointestinal changes, and the physiologically distinct functions of the digestive system are affected by aging.

## 3. The gut-brain axis

The gut-brain axis involves bidirectional communication between the central nervous system (CNS) and the enteric nervous system (ENS), primarily through the vagus nerve, neurotransmitters, and the gut microbiota. It regulates digestion, immune responses, and even mood and cognition. Disruptions in this axis are linked to gastrointestinal disorders, neurodegenerative diseases, and mental health conditions [14]. The nervous system plays a significant role in the decline of the digestive system in aging people. Aging affects both the central and autonomic nervous systems, particularly the ENS, which directly controls gut motility, secretion, and blood flow. Degeneration of enteric neurons and a decline in neurotransmitter activity, such as reduced acetylcholine levels, lead to slower peristalsis, delayed gastric emptying, and reduced coordination of intestinal motility [4]. Additionally, a decrease in vagal nerve function disrupts the gut-gut-brain axis, further slowing digestion and reducing the efficiency of nutrient absorption [14]. These changes cause common gastrointestinal issues in elderly people, including constipation, dysphagia, gastroparesis, and increased sensitivity to infections owing to weakened gut immunity. Moreover, reduced natural sensory processes from the gut to the brain may impair appetite regulation, leading to nutritional deficiencies. Digestive decline is sped up by ongoing, mild inflammation and damage from oxidative stress in the nervous system. These findings indicate that there is a strong connection between the aging of the nervous system and problems related to GI functions in elderly individuals, which has a strong impact on their health and quality of life [4].

## 4. The Gut Microbiota and Its Role in the Digestive Function of Elderly Individuals

### 4.1 The gut microbiota

The gut microbiota (GM) is a highly complex ecosystem that plays vital roles in digestion, metabolic regulation, immune function, and overall health, and in elderly people, greater microbial diversity may be particularly important for maintaining digestive health. The gut microbiome, which comprises bacteria, viruses, fungi, and archaea, contributes significantly to healthy aging by supporting essential gut functions, maintaining intestinal barrier integrity, defending against pathogenic organisms, and facilitating the metabolism of nutrients and pharmaceutical compounds [15,16]. Age-related dysbiosis in the host microbiota is characterized by alterations in microbial composition, function, and diversity, where aging is commonly associated with a decline in overall microbial diversity, a diminished abundance of key commensal taxa, and the emergence or increased presence of pathobionts that replace beneficial microbial populations [17]. During dysbiosis, certain pathogenic microbial metabolites can translocate into the bloodstream and function as signaling molecules in host microbiota interactions, where they influence and modulate immune, metabolic, and neurological pathways, often contributing to systemic inflammation [15].

### 4.2 Gut microbiota composition in elderly people

The GM composition of elderly people may differ from that of young adults living in the same area and having similar eating habits [18,19]. The GM of elderly individuals shows more variation than that of younger adults [20]. The gut microbiota is proposed as a key candidate marker of healthy aging, and centenarian microbiota composition has been repeatedly used as a healthy aging model [21]. Biagi et al. (2010) reported that elderly people exhibit a distinct and unique GM composition compared with young adults (approximately 30 years old) and elderly individuals (approximately 70 years old), who share a similar microbiota structure. In both younger groups, Bacteroidetes and Firmicutes dominated, whereas Actinobacteria and Proteobacteria were present in smaller proportions, with comparable microbial diversity. Notably, neither microbiota composition nor diversity shows a linear relationship with age [22]. Another study by Biagi analyzed the gut microbiota of semisupercentenarians (aged 105-109) in adults, elderly individuals, and centenarians in 2016. Thus, researchers have reported a decrease in the abundance of the core microbiota of symbiotic bacteria of the families Lachnospiraceae, Ruminococcaceae, and Bacteroidaceae and an increase in the abundance of opportunistic bacteria with age. However, a distinctive characteristic observed mainly in semisupercentenarians was the enrichment of health-associated bacteria, including *Akkermansia*, *Bifidobacteria,* and Christen-senellaceae, which are known for their roles in immunomodulation and metabolic homeostasis [19]. The authors speculated that these specific bacterial taxa may contribute to a new state of homeostasis in the aging host, potentially supporting longevity [19]. Aging is associated with a decrease in gut microbiota diversity and the growth of opportunistic species, including *enterobacteria, enterococci, staphylococci,* and *streptococci*. Firmicutes and Bacteroidetes populations have also been observed, accompanied by a decline in short-chain fatty acid (SCFA) production, particularly that of butyrate [23]. Rampelli et al. demonstrated that aging alters the gut microbiome profile, leading to a loss of genes involved in SCFA production, characterized by a decrease in saccharolytic bacteria and an increase in proteolytic bacteria. This shift is accompanied by the presence of pathobionts, which are associated with inflammation [24]. Additionally, higher levels of the mucin-degrading bacterium *Akkermansia muciniphila* were detected in older individuals than in young adults. Along with these changes in the gut microbiota, Elderly people presented high inflammation scores, supporting the inflammation hypothesis [22]. In addition to a study carried out in Europe, Wang et al. studied the gut microbiota of centenarians (100~108 years) and young elderly individuals (85~99 years) from Bama County, Guangxi, China, and elderly individuals (80~92 years) from Nanning city, Guangxi, China. Importantly, centenarian gut communities were highly enriched with *Roseburia* and enriched with *Escherichia* with depletion of *Lactobacillus*, Faecalibacterium, and *Akkermansia*. Overall, older people presented greater gut microbiota diversity than younger elderly individuals did. Additionally, the study revealed significant structural changes in butyrate-producing bacteria within the Firmicutes phylum and a more frequent presence of Bacteroidetes in elderly people than in their younger counterparts from the same region [25]. However, it is important to note that the diet of Chinese people is largely based on rice and plant-based foods, which may promote a more balanced GM structure, contributing to health maintenance in elderly people. Ultimately, the authors suggested that both age and a high-fiber diet could foster a newly balanced gut microbiota, which may play a key role in supporting health in elderly people [25]. Tuikhar and colleagues examined gut and fecal samples from elderly people (aged 100 years) and compared them to those from younger individuals (aged 25-45 years) in a region in India known for having many centenarians. In addition, these findings were compared with those of similar groups from Italy, Japan, and China, which included 125 centenarians. Overall, the authors reported large variations in bacterial diversity among centenarians in the four countries. In particular, elderly people have a large variety of species in the family Ruminococcaceae [26]. Notably, one unnamed species in the Ruminococcaceae family is Ruminococcaceae D16, which aids healthy immune responses to fight inflammation and gradual aging. Additionally, the authors described a decrease in the abundance of *Faecalibacterium*, a putative butyrate producer associated with inflammation. In the Bacteroidetes phylum, *Porphyromonaceae and Rikenellaceae* (Alistipes) (including *Porphyromonas, Odoribacter*, and *Parabacteroides*), as well as butyrate producers, were found to be more abundant in all elderly people [26]. Overall, elderly people often harbor a unique microbiome that differs from those of younger adults and even elderly individuals in general. Some studies using molecular techniques to analyze the gut microbiota in centenarians are shown in (Table 1).

**Table 1:** Studies utilizing molecular techniques to examine the gut microbiota composition in elderly people.

Subjects and age	Country	Alteration of Gut Microbiota	Molecular technique	Ref.
Elderly 73-101y	United Kingdom	↑Enterobacteriaceae	16S rRNA qPCR	[27]
Centenarians (C), Italy, 99-104y (14F, 1 M)	Italy	↑*Eggerthella*, *Christensenellaceae*, *Akkermansia*, *Bilophila*, and *Synergistaceae*, ↓*Bacteroidaceae*, *Ruminococcaceae*, *Anaerotruncus*, *Lachnospiraceae*, *Faecalibacterium*, *Coprococcus*, and *Roseburia*.	16S	[19]
Centenarians	Italy	↑Methanobrevibacter smithii (pathobionts); ↓SCFA producers	16S	[21]
	Italy	↓SCFA, ↑*Methanobrevibacter smithii* and *Akkermansia muciniphila*, lipopolysaccharide (LPS)	Shotgun	[28]
Healthy elderly	Japan	↓*Akkermansia*, *Christensenellaceae*, and *Butyricimonas*.	16S	[29]
Newborn to Centenarians	Japan	↑*Odoribacter*, *Butyricimonas*, *Christensenellaceae*, *Lactobacillus*, *Oscillospira*, *Oxalobacter*, and *Butyrivibrio*, SCFA-producing bacteria and *Bifidobacterium*.	16S	[30]
Centenarians (C)., 99-104y (20F, 1 M)	Italy	↓Bacteroides, Bifidobacteria, and overall species diversity, ↑Clostridia, Proteobacteria, *Eubacterium limosum,* and *Bacilli.*	HITChip, 16S rRNA qPCR	[22]
Institutionalized elderly,77-95y	Spain	↓Bacteroides, *Clostridium Faecalibacterium*, and cluster XIVa in elderly individuals, ↑L*actobacillus* group.	16S rRNA qPCR	[23]
Group RC, 92-108y	China	↑ *Escherichia* and Roseburia in centenarians, ↓ Faecalibacterium, Mitsuokella, Megamonas, *Lactobacillus*, Butyricimonas, Parabacteroides, Sutterella, Caprococcus, and *Akkermansia* in centenarians.	16S rRNA gene sequencing	[31]
	China	An increase in alpha diversity was noted, along with elevated levels of *Akkermansia* and *Christensenellaceae*.	16S	[32]
	China	↑*Akkermansia*, *Parabacteroides*, *Paraprevotella*, ↓core short-chain fatty acid (SCFA) producers declined.	16S	[33]
Centenarians	China	↑*Butyricimonas*, *Butyricicoccus*, *Odoribacter*, *Alistipes*, *Christensenella*, *Barnesiella*, and other pathobionts, ↓ SCFA producers and *Megamonas*	16S	[18]
	China	↑*Lactobacillus salivarius*, *Butyrivibrio crossotus*, *Subdoligranulum variabile*, *Roseburia hominis*, *Coprococcus catus*, *Clostridium sachharolyticum*, *Akkermansia muciniphila*, and *Victivallis vadensis*, ↓*Bifidobacterium*	Shotgun	[34]
Institutionalized elderly, 78-94y	Austria	↑Bacteroides in elderly and ↓ *Clostridium, Bifidobacterium*, cluster IV, total bacteria and *Clostridium* cluster IV diversity in elderly	16S rRNA qPCR, PCR-DGGE,	[35]
Centenarians	South Korea	↑*Akkermansia* and *Christensenellaceae*, ↓SCFA-producing bacteria and *Prevotella*	16S rRNA gene sequencing	[36]
	South Korea	There was an increase in *Prevotella copri*, pathobionts, and *Roseburia inulinivorans*, while core SCFA producers, *Megamonas*, *Alistipes*, *Bacteroides uniformis*, and *Bacteroides vulgatus* declined.	16S rRNA gene sequencing	[37]
Elder People	USA	Aging was associated with increased gut microbiome uniqueness (or dissimilarity) and reduced *Bacteroides* abundance, with a more rapid shift toward uniqueness observed in the healthy aging group.	16S	[17]
Centenarians	India	↑*Akkermansia*, *Butyricimonas*, and *Ruminococcaceae*, ↓*Prevotellaceae* and core SCFA	16S	[26]
Elder people	Thailand	↑*Eubacterium eligens*, *Bacteroides thetaiotaomicron*, *B. uniformis*, *B. caccae*, *B. ovatus*, ↓ *Parabacteroides distasonis* SCFA-producing bacteria.	16S	[38]

### 4.3 Summary of gut microbiota changes by country in elderly people

In elderly people across various countries, distinct alterations in the gut microbiota have been observed. Italy and Japan show increased pathobionts such as *Akkermansia* and *Christensenellaceae,* with a decline in SCFA producers. Chinese and Korean centenarians present elevated levels of multiple pathobionts and reduced diversity of beneficial microbes. The U.S. reported greater microbiome uniqueness with lower *Bacteroides*, while countries such as India, Thailand, and Austria also presented increased harmful microbes and decreased beneficial microbes, indicating a global pattern of gut dysbiosis with aging, as shown in (Table 2).

**Table 2:** Summary of gut microbiota changes by country in elderly people.

Country	Microbiota Changes
Italy	↑ *Eggerthella*, *Christensenellaceae*, *Akkermansia*, *Bilophila*, *Synergistaceae*, *Methanobrevibacter smithii* ↓ *Bacteroidaceae*, *Ruminococcaceae*, core SCFA producers, *Faecalibacterium*, *Roseburia*, *Coprococcus* ↑ Xenobiotic degradation, LPS biosynthesis, phenolic metabolites
Japan	↑ *Akkermansia*, *Christensenellaceae*, *Butyricimonas*, *Odoribacter*, *Lactobacillus*, *Oscillospira*, *Oxalobacter*, *Butyrivibrio* ↓ Core SCFA producers, *Bifidobacterium*
China	↑ *Akkermansia*, *Parabacteroides*, *Paraprevotella*, *Butyricimonas*, *Odoribacter*, *Christensenellaceae*, *Butyricicoccus*, *Alistipes*, *Barnesiella*, *Lactobacillus salivarius*, *Victivallis vadensis* ↓ Core SCFA producers, *Megamonas*, *Prevotella*, *Bifidobacterium*, *Faecalibacterium*, *Sutterella*, *Caprococcus*
South Korea	↑ *Akkermansia*, *Christensenellaceae*, *Prevotella copri*, *Roseburia inulinivorans* ↓ *Prevotella*, *Megamonas*, *Alistipes*, *Bacteroides uniformis*, *B. vulgatus*, core SCFA producers
USA	↑ Microbiome uniqueness/dissimilarity ↓ *Bacteroides*
India	↑ *Akkermansia*, *Butyricimonas*, *Ruminococcaceae* ↓ *Prevotellaceae*, core SCFA producers
Thailand	↑ Pathobionts including *Eubacterium eligens*, *Bacteroides thetaiotaomicron*, *B. uniformis*, *B. caccae*, *B. ovatus*, *Parabacteroides distasonis* ↓ Other core SCFA producers
Spain	↑ *Lactobacillus* group ↓ *Bacteroides*, *Clostridium*, *Faecalibacterium*, cluster XIVa
Austria	↑ *Bacteroides* ↓ *Clostridium*, *Bifidobacterium*, cluster IV diversity

### 4.4 Factors affecting changes in the GM with aging

The composition of the GM can influence the aging process, with several factors, such as dietary habits, lifestyle, and medication use, contributing to age-related changes. As individuals grow older, shifts in diet, including reduced fiber intake and increased consumption of processed foods, can negatively affect microbial diversity. Additionally, aging is often accompanied by changes in immune function, altered gut permeability, and increased chronic low-grade inflammation, which can disrupt the balance of the microbiome [3]. The GM is described by Biagi et al. as a dynamic organ that maintains a functional relationship with the host rather than acting as a dysbiotic or nonfunctioning organ. A major challenge is to identify those factors that enhance health throughout life, since aging is associated with different diseases [23]. In elderly individuals, infectious diseases, antibiotic use, and the consumption of various medications can lead to harmful changes in the gut microbiome, known as dysbiosis, which often contributes to age-related diseases [39]. The diet is very influential in the GM; those who consume too much fat, mainly saturated fats, tend to experience higher levels of bacterial endotoxins in their blood. Because of this increase in endotoxins, endotoxemia occurs and causes constant, mild inflammation throughout the body. Changes in eating habits make the GM imbalanced, add to aging and lead to a greater risk of different types of health concerns [40]. Additionally, changes in the intestinal nervous system and alterations in the GM are commonly observed [41,42]. In another study, Wang et al. explored poor dietary habits, such as a low intake of fruits and vegetables, that can affect the balance of the GM. The authors also reported that a high-fiber diet promotes a stable GM population, which could help maintain health in the elderly population [25].

The relationship between the gut microbiota and digestive function in elderly individuals requires a clear distinction between observational correlation and mechanistic causality. While many studies report associations between microbial shifts and age-related digestive decline, these findings alone do not prove causation [43]. A correlation between reduced microbial diversity and slower intestinal transit in older adults may reflect broader aging processes rather than a direct microbial effect. Mechanistic insights, in contrast, involve experimental validation of how the gut microbiota actively regulates digestive physiology. Studies using germ-free animal models have demonstrated that microbiota transplantation from aged donors can impair gut motility, suggesting a causative role [44]. Additionally, microbial metabolites such as SCFAs enhance intestinal barrier function and stimulate smooth muscle contractions, directly linking bacterial activity to digestive efficiency. Nutritional interventions further help differentiate correlations from mechanisms. While observational data may link high-fiber diets to better digestion, randomized controlled trials have shown that specific prebiotics increase *Bifidobacterium* abundance and improve bowel regularity, confirming a microbiota-mediated effect [45].

## 5. The gut-associated immune system and digestive health in aging

The gut-associated immune system (GAIS) plays a fundamental role in supporting the digestive health of aging people, as changes in both immunity and gastrointestinal function can contribute to disease susceptibility. GM-derived metabolites such as SCFAs, polyamines, and tryptophan-derived metabolites are associated with host metabolism, mitochondrial function, and aging-associated inflammation. Age-related gut dysbiosis reduces the levels of beneficial metabolites such as SCFAs, polyamines, and anti-inflammatory tryptophan catabolites while increasing the levels of proinflammatory compounds such as LPS and secondary bile acids. This imbalance disrupts epithelial barrier integrity, activates immune pathways, and promotes systemic inflammation. The resulting oxidative stress and mitochondrial dysfunction contribute to chronic inflammation and cellular senescence, accelerating the aging process.

### 5.1 Inflammaging and immune-microbial interactions

Dysbiosis of the gut microbiota enhances intestinal permeability, also known as "leaky gut," allowing opportunistic infections and their microbial metabolites or toxins to infiltrate the circulation, thereby prompting an inflammatory response. Consequently, immunological responses facilitated by the gut microbiota are crucial for maintaining intestinal integrity and preventing increased permeability [46]. The proteins that constitute intestinal epithelial cells (IECs) are occludin, claudin, and junctional adhesion molecules (JAMs), which regulate the paracellular transfer of ions and substances. Together, changes in or damage to these tight junction proteins that hold the cells in the intestinal barrier disrupt the integrity of the barrier, which leads to increased intestinal permeability, allowing harmful substances such as toxins, pathogens, and undigested food particles to interact with the immune system and cross the barrier [4]. Stimulation of permeability can stimulate immune cells, such as dendritic cells and macrophages, activate inflammatory responses, enhance antigen presentation, and possibly cause autoimmune disease. Altered permeability may modify the survival and proliferation of microbial populations in the context of modifications in the composition of the gut microbiota. This can lead to a vicious cycle of inflammation as well as impairment of the intestinal barrier and the production of proinflammatory cytokines. A healthy intestinal barrier, which is maintained by intact tight junction proteins, adopts a more tolerant immune environment through the prevention of excessive microbial translocation, limiting immune activation, supporting a balanced microbiota, maintaining immune homeostasis, and promoting anti-inflammatory cytokine synthesis [4,14]. During aging, intestinal barrier function decreases gradually with subsequent age-related dysbiosis and increased intestinal permeability, leading to local mucosal inflammation. This, in turn, allows bacterial endotoxins to enter the bloodstream, triggering systemic inflammation [47].

Gut-associated lymphoid tissue (GALT) is a critical component of the immune system located in the gastrointestinal tract. It defends against pathogens while maintaining tolerance to beneficial microbes and dietary antigens. However, these microorganisms are inconvenient, as they are crucial for maintaining immune balance by communicating with neurons of the gut-associated GALT, an essential part of the immune system. Some of the specialized immune cells in GALT are macrophages, dendritic cells, and T and B lymphocytes, which help differentiate harmful and harmless microbes [4]. Studies have shown that early-life microbial colonization influences immune development, and deviations in this process have been linked to an increased risk of autoimmune diseases and allergies [48]. SCFAs are microbial metabolites, mainly acetate, propionate, and butyrate, produced by gut bacteria through fermentation of dietary fiber. They support gut health by nourishing colon cells, maintaining barrier integrity, and modulating inflammation. These SCFAs play crucial roles in modulating immune responses by promoting anti-inflammatory pathways and enhancing regulatory T-cell (Treg) differentiation, which helps maintain immune tolerance and prevents excessive immune activation [4].

The intestinal mucosal barrier, which is the body's largest immune defense, is significantly impacted by the aging process [49]. Research has indicated that the quantity of intestinal immune cells remains stable with age, but their functional capacity decreases as people age [50]. Thevaranjan et al. reported that, compared with those from younger individuals, macrophages from older individuals exhibited a notably reduced bactericidal ability. Furthermore, similar findings have also been reported for dendritic cells (DCs) and natural killer (NK) cells [50]. In addition, senescence affects primarily T-cell immune function, essentially by controlling the proliferative response of Peyer's patches in CD4+ T cells. Both the reduced appearance of immunoregulatory molecules in LP CD4+ T cells and the reduction in the occurrence and function of LP CD4+ and Th17 cells contribute to the disruption of the mucosal barrier and altered perception of the gut microbiota [49].

**Figure 2.**
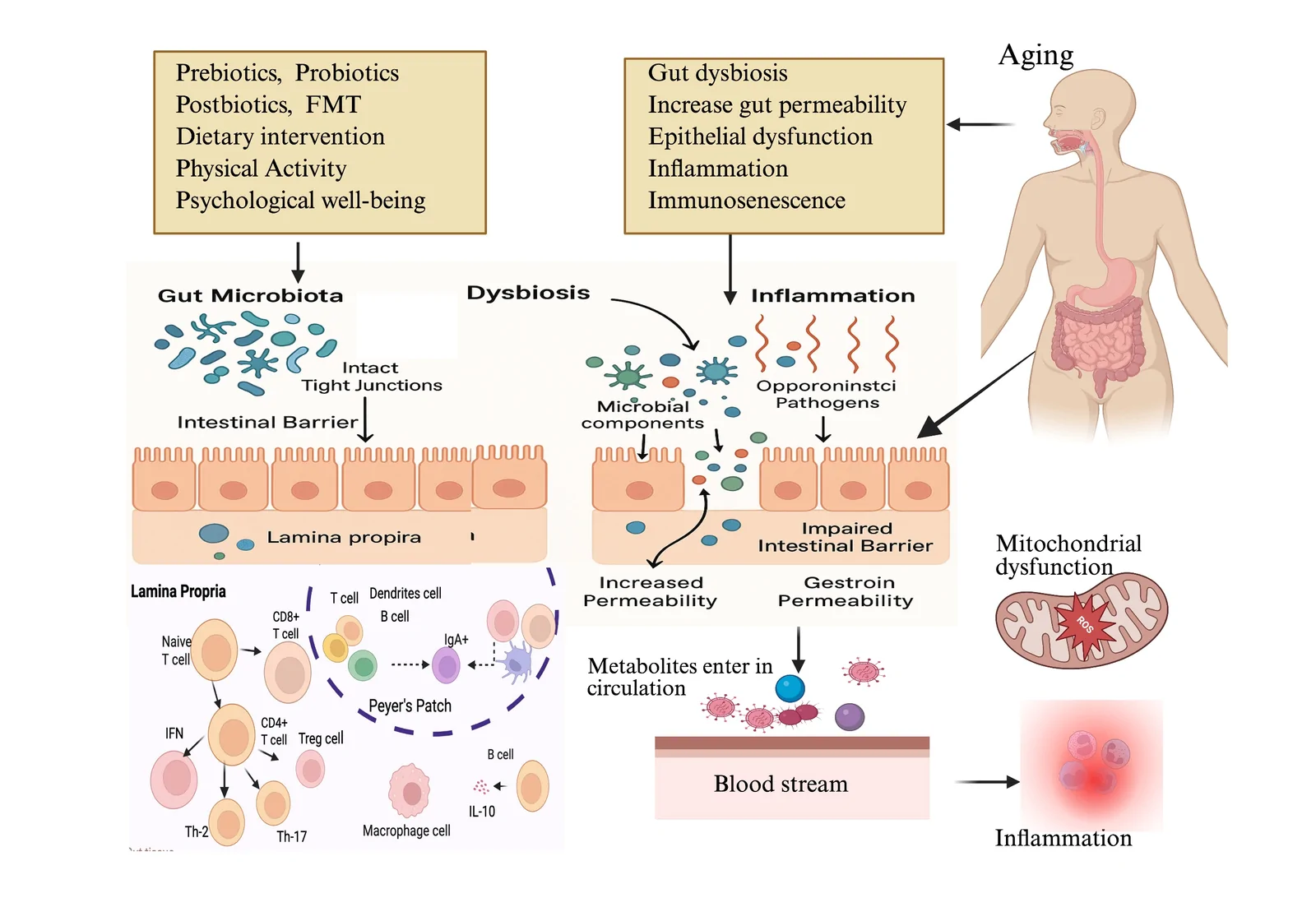
**This figure describes the impact of aging on the gut microbiota and intestinal health**. Aging-related factors such as tooth loss, stress, and reduced sensory functions contribute to gut microbial dysbiosis, leading to epithelial dysfunction and increased intestinal permeability. This allows microbial components and metabolites to enter the circulation, triggering systemic inflammation. The inflammatory response and mitochondrial dysfunction further contribute to digestive decline and overall health deterioration. Potential interventions such as probiotics, dietary changes, and physical activity may help maintain gut health. Created by Biorender.

The gut-immune system interaction is not only confined to localized immune responses but also influences systemic immunity and different disease pathologies [51]. Specific bacterial strains have been identified as modulators of immune function, either by directly stimulating immune cells or by producing metabolites that regulate inflammation. For example, *Faecalibacterium prausnitzii,* a beneficial gut bacterium, has been associated with anti-inflammatory effects through its production of butyrate, which helps reduce colonic inflammation and supports epithelial barrier integrity [52]. Pathogenic bacteria such as *Escherichia coli* and *Clostridium difficile* can disrupt immune homeostasis, leading to chronic inflammation and disease progression. Additionally, gut microbiota influences the efficacy of immunotherapies, particularly in cancer treatment. Recent studies have shown that specific gut microbial compositions enhance responses to immune checkpoint inhibitors, a class of cancer immunotherapy drugs [53]. Aging alters the gut microbiota through factors such as tooth loss, stress, and impaired digestion, leading to dysbiosis, increased gut permeability, systemic inflammation, and mitochondrial dysfunction; however, probiotics, diet, and exercise can help restore gut health, as shown in (Fig. 2).

### 5.2 Mitochondrial dysfunction

Aging is associated with mitochondrial dysfunction, which is characterized by a gradual decline in mitochondrial performance over time. This decline is associated with age-related conditions such as digestive issues; neurodegenerative diseases such as Alzheimer’s disease and Parkinson’s disease; cardiovascular diseases; diabetes; obesity; and even cancer [54]. Mitochondria play crucial roles in energy production, and their dysfunction leads to reduced cellular energy and impaired cellular repair mechanisms. Over time, this contributes to the deterioration of tissues and organs, accelerating the aging process and increasing vulnerability to chronic disease [55]. The buildup of mitochondrial DNA mutations leads to increased oxidative stress in the mitochondria and subsequent mitochondrial dysfunction through the production of reactive oxygen species (ROS) [56]. In the gut, the induction of senescence can impair oxygenation of the intestinal epithelium and cause mitochondrial dysfunction, which then drives inflammation and, in turn, alters the composition of that microbial community, triggering the onset and subsequent escalation of senescence-induced inflammation [57]. In addition to shaping mitochondrial function and metabolism in intestinal cells, the gut microbiota can also do so via bacterial metabolites, namely, SCFAs and secondary bile acids [58]. Dysbiosis reduces the production of key metabolites such as SCFAs, polyamines, indole derivatives, and urolithins, which are vital for maintaining gut integrity and mitochondrial health, and this disruption impairs mitochondrial metabolism, increases intestinal oxygen levels, promotes the growth of pathogenic bacteria, and elevates proinflammatory compounds such as lipopolysaccharides and secondary bile acids, thereby accelerating oxidative stress, chronic inflammation, cellular senescence, and the progression of age-related diseases.

### 5.3 SCFAs

The host, gut microbiota, and diet interact to produce bioactive molecules that regulate immune function, gut integrity, and tissue maintenance. However, aging reduces metabolite production, weakens the gut barrier, increases inflammation, and increases the risk of immune disorders [59]. Research indicates that the levels of SCFAs, including acetate, propionate, and butyrate, generally decrease with age [60]. People present higher SCFA levels in their fecal samples, along with a gut microbiota that possesses enhanced glycolytic capacity [21]. Altering the gut microbiota of older mice by transplanting fecal material from young mice increased their life expectancy without causing inflammation or damage to the intestinal wall. Many of these benefits are attributed to *Akkermansia muciniphila* and the acetic acid it produces [61]. These findings indicate that SCFAs have a strong effect on aging. With aging, the number of SCFAs produced in the gut decreases, which can be harmful to the health of the gut and increase the vulnerability of the intestines to infections and common diseases associated with aging [60]. Additionally, it enhances the anti-inflammatory functions of colonic macrophages and dendritic cells (DCs) by activating GPR109a signaling, promoting the differentiation of regulatory T cells (Tregs) and IL-10-producing T cells [62]. GPR41 and GPR43 receptors are activated by the short-chain fatty acids acetate and propionate, initiating immune responses in epithelial cells through the ERK1/2 and p38 MAPK signaling pathways. These short-chain fatty acids help maintain gut homeostasis and support immune function, thereby reducing the risk of chronic inflammation and promoting overall health [63].

### 5.4 Polyamines

Recent research has revealed that polyamine metabolism plays a vital role in the aging process, with spermine, spermidine, and putrescine being the most extensively studied polyamines. They are important for DNA repair, cell growth, and the regulation of apoptosis. A decrease in polyamine levels after we age can change how our cells function, which can accelerate aging and lead to age-related disease [64]. In rodents, the total levels of putrescine and spermidine decrease with age, whereas brain-specific concentrations of spermine decrease with age [65]. The results from Pucciarelli and others show that in whole blood, polyamine levels first decrease as people get older but later seem to rise again. Older people aged 90 years had levels of spermine and spermidine similar to those reported in people under 50 years but higher than those reported in those aged 60-80 years. Emerging evidence indicates that increased dietary intake of spermine may contribute to a reduction in overall mortality, including deaths related to cardiovascular conditions [66]. This study offers compelling evidence that polyamines are closely associated with the aging process. In addition to being endogenously synthesized, polyamines are obtained through dietary sources and are also synthesized by the gut microbiota. While polyamines from food are primarily absorbed in the upper intestine, the majority of those present in the colon are produced by gut microbes. These findings highlight the critical role of the gut microbiota in regulating polyamine levels, which may significantly influence aging and the development of age-related health conditions [67].

### 5.6 Tryptophan

Microbiota-derived tryptophan (Trp) derivatives, including indole propionic acid (IPA), tryptamine, and indole, are closely linked to host aging and show promise in preventing age-related diseases. Moreover, tryptamine and its derivatives promote the protection of neuronal cells and inhibit IPA and neuronal damage, reducing swelling and neuronal apoptosis through the GPR30/AMPK/SIRT1 pathway, which protects against neurodegeneration [68,69]. Rampelli et al. reported that the GM of elderly people presented increased Trp metabolism-related genes [24]. Conversely, the indole content and TnaA abundance were lower in the feces of older individuals than in those of younger individuals. The effects of indole have been investigated in *Cryptomeria elegans, Drosophila melanogaster*, and mice, which suggests that indole extends a healthy lifespan without altering the maximum lifespan. Trp metabolism involves host-microbe cometabolism, which generates microbial-, host-, and shared-derived metabolites. *Lactobacillus* spp., *Bifidobacterium* spp., and *Clostridium anomalum* are specific bacterial species that produce the Trp metabolites indole-3-carboxaldehyde (ICA) and indole-3-acetic acid [70]. IPA enhances intestinal barrier integrity through the TLR4 and PXR receptors [71]. Since aging disrupts the gut microbiota balance, damages the gut mucosa, and promotes inflammation, Trp derivatives may counteract aging by modulating gut barrier function and immune responses, making them potential therapeutic agents for age-related health decline.

### 5.7 Bile acids

Bile acid metabolites play crucial roles in aging by influencing the gut microbiota, metabolism, and inflammation. Secondary bile acids, which are produced by gut bacteria, regulate key signaling pathways such as FXR and TGR5, impacting lipid metabolism and cellular stress responses. Dysregulation of bile acid homeostasis is linked to age-related diseases such as metabolic disorders and neurodegeneration. Studies suggest that bile acids modulate longevity by affecting mitochondrial function and immune responses. Targeting bile acid metabolism could offer therapeutic strategies for healthy aging [72].

## 6. Genetic Factors

### 6.1. Longevity-associated genes

The genetic makeup of elderly people has revealed several gene variants associated with longevity, including those involved in metabolic regulation, inflammation, and cell repair. These genetic factors may help support digestive function by reducing age-related damage to the GI tract and enhancing digestive resilience. Previous studies have shown that genetic factors have a major influence on how long a person lives; thus, researchers have studied which genes, and their variations affect the normal functions of cells and the body. Genes involved in stress, the immune system, and the way energy is used are very important in this process. Through GWASs, scientists have discovered that older people’s genetics include extraprotective single-nucleotide polymorphisms (SNPs) [73]. MUC2 is a gene in the intestinal mucus that shields epithelial cells from harmful bacteria and keeps the gut healthy. Recent studies have indicated that MUC2 is important for keeping microbes on the jejunum, which improves protection from infections. An increase or decrease in MUC2 expression and glycosylation is related to inflammatory bowel diseases such as ulcerative colitis [74]. Among the most significant longevity-associated genes are APOE, GHR, and the recently identified LAV-BPIFB4 [75]. These genes are involved in critical biological pathways affecting lifespan, including IGF-1, mTOR, and immune system regulation [76]. The APOE gene, which is critical for lipid metabolism, influences neurodegenerative and cardiovascular health. ε2 has three isoforms, ApoE ε2, ε3, and ε4, where ε2 is linked to increased lifespan, whereas ε4 is associated with age-related diseases [77]. D3-GHR deletion in the GHR gene increases the ability of cells to withstand oxidative stress, which reduces ROS and can increase longevity [78]. The recently identified LAV-BPIFB4 variant has been strongly linked to longevity. It is more common in older individuals and is associated with reduced inflammation, improved immune function, and a lower risk of cardiovascular and neurodegenerative diseases. Its recessive inheritance pattern may offer a genetic advantage in populations with higher rates of consanguinity, where long-lived individuals are more prevalent. In contrast, the RV-BPIFB4 variant has been associated with increased frailty and cardiac dysfunction [75]. Older individuals have genetic strength with these protective genes that confer resistance to aging-related diseases. The special arrangements of their genes act as examples of healthy aging and illustrate the influence of some genes on lifespan. The main genetic influences on human longevity include key genes such as APOE, GHR, and BPIFB4, which regulate processes such as stress resistance, metabolism, immune function, and cellular repair, contributing to increased lifespan and protection against age-related diseases.

## 7. Lifestyle Factors and Their Impact on Digestive Function

### 7.1 Physical Activity and Digestive Health

Physical activity plays an important role in the digestive health of elderly people, contributing significantly to their longevity and overall health. Regular walking helps maintain a healthy weight, improves blood circulation, and enhances the functioning of the gastrointestinal system by promoting regular bowel movements and preventing constipation [79]. Regular physical activity is important for preventing the development of cardiovascular disease, diabetes and disorders of the gastrointestinal system caused by aging. A healthy gut contributes significantly to overall digestive health, which is often observed in centenarians who remain active and engage well into old age. Therefore, incorporating physical activity into daily life is essential for maintaining gut health and supporting longevity [80].

### 7.2 Psychological well-being and digestive function

Psychological well-being plays an important role in the digestive function of aging people, as mental health is closely linked to gut health. Positive emotional states, such as happiness, resilience, and social engagement, have been shown to reduce stress, which in turn lowers the production of stress hormones that can negatively affect digestion [81]. For centenarians, maintaining a sense of purpose and strong social connections can alleviate anxiety and depression, both of which are known to disrupt gut function. Stressful feelings and chronic stress can worsen the gut microbiota and, in the long run, can lead to disorders of the digestive system, such as irritable bowel syndrome (IBS) and inflammation. Additionally, mental well-being leads to a better appetite and nutrient absorption, which also reduces gastrointestinal discomfort. Overall, the relationship between psychological health and digestive function is crucial for the longevity and vitality of centenarians [82].

## 8. Therapeutic strategies enhancing gut health to support healthy aging and longevity

Several therapeutic strategies have been recognized for their effectiveness in promoting longevity. These include caloric restriction, which slows aging and enhances lifespan, as well as the use of pharmacological agents. Lowering the body temperature of the gut microbiome (GM) through dietary interventions, probiotics, prebiotics, synbiotics, and fecal microbiota transplantation (FMT) has emerged as a promising strategy. A well-balanced GM contributes to metabolic regulation, boosts immune function, reduces inflammation, and enhances resilience against age-related decline.

### 8.1 Impact of diet innervation on aging

Diet plays a crucial role in shaping the intestinal microbiota and promoting healthy aging. Certain dietary patterns, such as caloric restriction (CR), are particularly effective in achieving this goal. Research has highlighted the benefits of plant-based, high-fiber diets, probiotics, and prebiotics in maintaining gut health. Polyphenols found in plant foods further contribute by enriching microbial diversity and strengthening gut metabolism. It is widely recommended that dietary fiber may help alleviate constipation in older adults, thereby regulating bowel movements and transit time [83].

**Figure 3.**
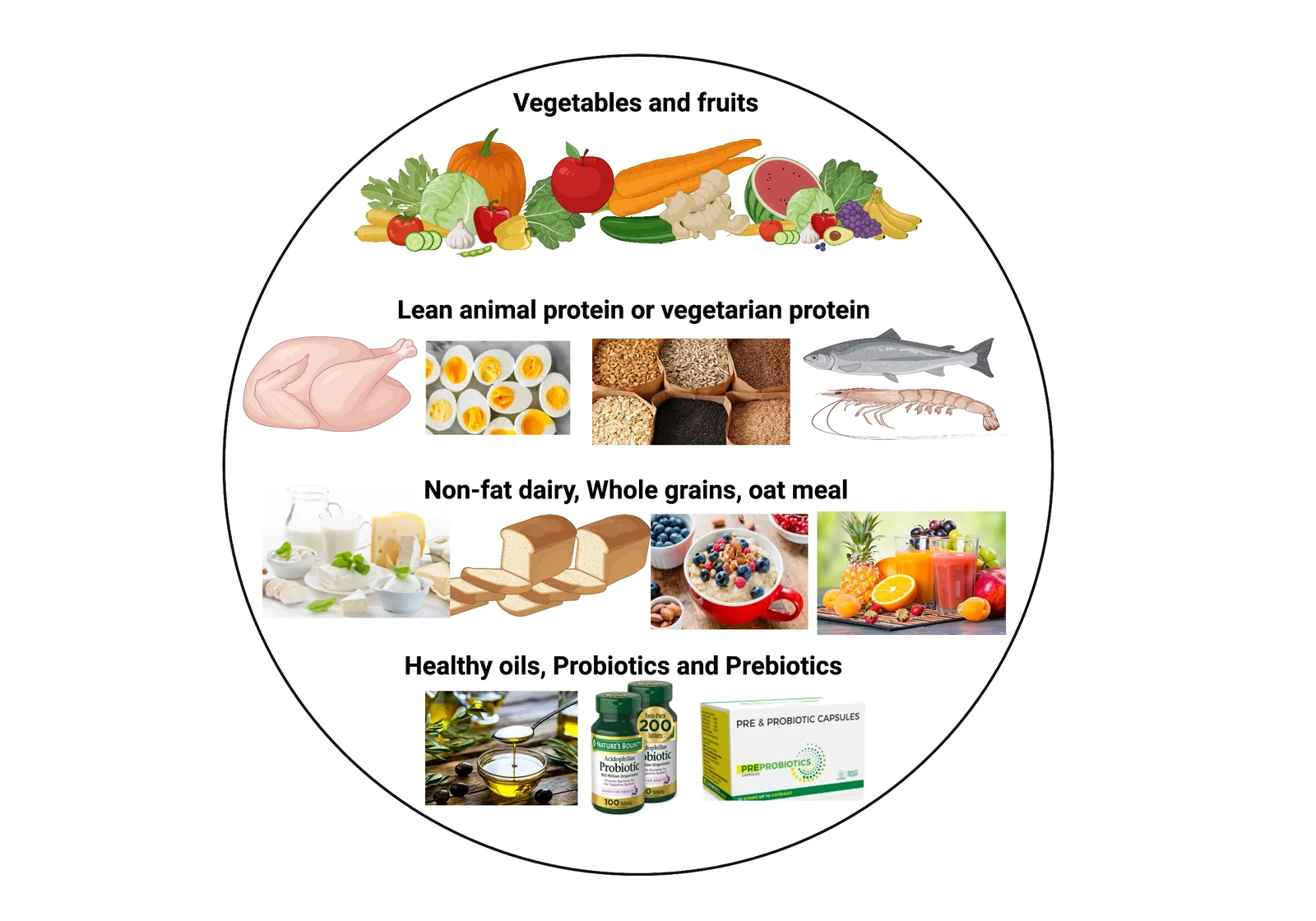
Diet intervention for the elderly people.

Elderly people’s diets typically consist of fiber-rich plant-based foods and minimally processed foods, which contribute to digestive health by promoting regular bowel movements and microbiota diversity while supplying essential nutrients such as polyphenols, vitamins, and minerals [84]. Fermented foods such as yogurt, miso and kimchi play vital roles in improving both the diversity of your microorganisms and how well your body digests. Elderly people eat less refined sugar and processed foods, which helps reduce their chances of metabolic disorders. Many medical practitioners include CR or intermittent fasting in their diet plans, as these methods have been shown to promote metabolic health and prolong life. Having an active lifestyle and eating with family and friends helps these ways of eating promote long-lasting health and a longer life [84]. Dietary choices significantly influence the gut microbiota composition, intestinal barrier integrity, and inflammation. The Mediterranean diet, which is rich in plant-based foods, is associated with a lower risk of chronic diseases, including cancer and cardiovascular diseases. The potential benefits may occur through microbiome-related changes, including higher levels of butyrate-producing bacteria and SCFAs in general, although the findings are mixed. Polyphenol-rich foods such as blueberries, green tea, and dark chocolate act as prebiotics, promoting beneficial microbes and increasing SCFA production. One study revealed that a diet rich in polyphenols improved gut health in elderly individuals by increasing beneficial bacteria and reducing serum zonulin, an indicator of intestinal permeability [85]. Figure 3 showed the diet intervention for the elder people.

### 8.2 Probiotics, prebiotics, and symbiotics

Probiotics have shown promising benefits for elderly individuals by supporting gut health, enhancing immune function, and potentially improving overall well-being. The most well-known probiotics include *Lactobacillus* and *Bifidobacterium*, which promote healthy aging by increasing beneficial gut bacteria and inhibiting harmful bacteria. Fang et al. reported that a probiotic combination containing *Lactobacillus casei SX-1107, Bifidobacterium animalis SX-0582*, *Bifidobacterium longum SX-1326*, and *Lactobacillus fermentum* SX-0718 improved cognitive and motor functions in aging mice [86]. In addition to *Clostridium* cluster IV, *Faecalibacterium prausnitzii,* XIVa, and *Christensenella minuta (C. minuta),* which are enriched in the intestines of elderly people, these bacteria play a common role in alleviating aging. They are SCFA-reducing bacteria that contribute to gut barrier protection through SCFA production, which in turn helps mitigate aging-related effects [87,88]. The abundance of beneficial bacteria, such as *Lactobacillus* spp. and *A. muciniphila,* is increased, and the abundance of opportunistic Proteobacteria spp. is reduced by *L. acidophilus DDS-1* [89]. *Lactobacillus paracasei PS23* has been found to delay the cognitive decline associated with aging in senescence-accelerated mouse prone 8 (SAMP8) mice, a model characterized by early-onset aging. This probiotic improved cognitive performance and reduced the progression of neurodegeneration. Through its influence on the gut microbiota, *L. paracasei PS23* shows promise as a potential approach for addressing cognitive decline in older individuals [90]. Christensenellaceae, a family within the phylum Firmicutes, is one of the five key taxa associated with a healthy gut. It is relatively highly abundant in elderly people and supercentenarians, serving as a microbial signature of longevity [91]. Since oxidative stress and inflammation are involved in aging and probiotics have antioxidant and immunomodulatory effects, it is reasonable to believe that probiotics can promote longevity [92].

Prebiotics are dietary fibers that selectively stimulate the growth and activity of beneficial gut bacteria, such as *Bifidobacteria* and *Lactobacilli*. In elderly individuals, GM diversity often decreases, leading to increased inflammation and weakened immune function. The most common prebiotics include galacto-oligosaccharides, inulin, lactulose, fructo-oligosaccharides, pectic-oligosaccharides, xylo-oligosaccharides, and trans-glucosylated oligosaccharides [93]. Some strains, such as LKM512 and *B. licheniformis*, are associated with increased longevity, whereas others help increase immunity and make the mind more active without directly influencing lifespan. Foods rich in inulin and polyphenols can improve gut health by strengthening good bacteria and diminishing inflammation [92,94]. Clinical studies have demonstrated that prebiotics improve bowel regularity and stool consistency in older adults, addressing common digestive issues such as constipation. Moreover, prebiotics enhance the absorption of minerals, especially calcium and magnesium, supporting bone health in aging populations [95]. Emerging research also highlights the potential role of prebiotics in cognitive health through modulation of the gut-brain axis by reducing neuroinflammation and influencing neurotransmitter synthesis. Overall, prebiotic supplementation is a promising, noninvasive strategy to counteract age-related gut dysbiosis and chronic inflammation, thereby promoting healthier aging and improved quality of life [96].

Synbiotics, a combination of prebiotics and probiotics, help regulate the composition of the gut microbiome by promoting the growth of beneficial bacteria. This improved microbial balance supports immune function and contributes to overall health. By strengthening immune responses and lowering inflammation, synbiotics play a role in supporting healthy aging and longevity [97]. The health benefits of postbiotics, such as SCFAs and polyamines, are stable and safe, helping strengthen the gut and the immune system. Since research has not shown much difference, studies should continue to determine if they truly have an impact on aging. Evidence from clinical studies in elderly individuals has revealed that synbiotics may lead to better bowel health, more regular bowel movements, and less bloating. Moreover, synbiotics have been associated with decreased levels of systemic inflammatory markers, such as C-reactive protein (CRP), and improved phagocytic activity of immune cells, thereby contributing to greater resistance against infections and chronic inflammation [98]. Table 3 shows key future directions for improving digestive health in elderly individuals through the gut microbiota and nutritional strategies. It highlights personalized therapies, dietary guidelines, optimized FMT, and gut-brain axis research as promising translational paths while also addressing challenges such as variability, regulatory issues, and the need for large-scale studies and technology integration.

**Table 3:** This table highlights key future research and clinical strategies to improve the digestive health of elderly individuals through personalized microbiome therapies, diet, and FMT while addressing practical challenges.

Future Direction	Description	Translational Potential	Challenges and Considerations
Personalized Microbiome-Based Therapies	Development of tailored probiotic, prebiotic, and synbiotic interventions based on individual gut microbiota profiles in elderly patients.	Improved digestive function and reduced age-related GI disorders through targeted microbiota modulation.	High interindividual variability; need for robust biomarkers and precision diagnostics.
Dietary Protocols for Gut Health in Aging	Designing and validating age-specific dietary guidelines that promote beneficial microbiota and digestive function, including fiber and polyphenol-rich diets.	Enhances nutrient absorption, reduces inflammation, and supports healthy aging.	Requires large-scale clinical trials; adherence and cultural dietary variations need addressing.
FMT (Fecal Microbiota Transplant) Optimization	Refinement of FMT protocols for safety, donor selection, and delivery methods specifically for elderly populations.	Potential to restore microbial diversity and improve gastrointestinal and systemic health.	Regulatory hurdles, risk of adverse effects, and long-term safety data are limited.
Gut-Brain Axis Interventions	Exploring microbial metabolites’ impact on neurogastroenterology and cognitive function in elderly individuals.	May offer new avenues for managing neurodegenerative diseases linked to gut dysbiosis.	Complex mechanistic pathways; need for interdisciplinary research integration.
Regulatory Framework Development	Advocating for clear guidelines on probiotics, prebiotics, and microbiota-based therapies in elderly care.	Facilitates safe, standardized clinical use and accelerates translation to practice.	Requires collaboration among researchers, clinicians, and regulatory bodies.

### 8.3 Fecal microbiota transplantation

FMT is emerging as a promising intervention to restore the gut microbial balance and improve GI health in elderly individuals, a population particularly susceptible to microbiota dysbiosis due to aging-related physiological changes, polypharmacy, and reduced dietary diversity. Studies have demonstrated that FMT can effectively treat recurrent *Clostridioides difficile* infection (rCDI) in older adults, with cure rates comparable to those in younger populations, often exceeding 80% efficacy [3]. In addition to rCDI, FMT has been demonstrated to reduce the symptoms of irritable bowel disease (IBD), IBS and antibiotic-associated diarrhea in seniors through restoring the gut’s microbial community, protective barrier, and release of anti-inflammatory compounds [99].

## 9. Conclusion and Future Perspectives

The digestive function of aging people has revealed key factors contributing to their longevity and digestive resilience, although much remains to be understood. Gnotobiotic and fecal transplant studies highlight the essential role of the microbiota and microbial metabolism in the health benefits of phytochemicals. Advanced technologies such as metabolomics are expected to reveal new microbial metabolites that support healthy aging. Expanding metabolomics databases and improving intestinal microbiota sampling will enhance our understanding of microbial metabolism. Future research will focus on metabolic pathways rather than specific taxa, as different microbes often share similar metabolic functions. Clinical trials are needed to confirm the bioactivity and health benefits of newly discovered microbial metabolites.

Genetics will also play a key role in understanding how centenarians maintain digestive function despite the physiological declines associated with aging. Advances in genomics help identify longevity-associated genetic variants that influence digestive resilience. While some genetic predispositions to longevity have been identified, the specific genes that help maintain gastrointestinal health over a long lifespan are still largely unknown. Future research should focus on the molecular mechanisms behind these genetic adaptations, examining how these genes maintain gut integrity, ensure nutrient absorption, and reduce systemic inflammation. The investigation of how lifestyle factors such as diet, exercise, and stress management affect the expression of longevity-related genes will also be important because these factors may facilitate optimum digestive health in older adults. This could lead to the development of genetic or pharmacological interventions to improve digestive function and increase health. The complex interactions among genetics, diet, microbiota, physical activity, and mental well-being highlight the need for interdisciplinary collaboration in areas such as genetics, nutrition, immunology, and psychology. Chronic low-grade inflammation, particularly in the gut, is a significant factor in digestive decline, and future research should explore how centenarians manage to maintain low inflammation levels and whether their unique gut microbiota, dietary habits, or lifestyle factors play a role in this process. Psychological factors, such as stress management, social engagement, and mental resilience, also influence digestive health and should be studied more deeply.

In conclusion, the digestive function of elderly people involves complex interactions with genetic, environmental, dietary, and lifestyle factors. Normal aging is supported by diet, lifestyle, the genetic makeup of an individual, and their gut microbes, which prevent common age-related illnesses. Eating phytochemicals can enhance gut health by lowering inflammation, helping different types of bacteria live there, and allowing our bodies to adapt well to changes in diet. These effects are important for maintaining a healthy gut and during the aging process. A balanced and diverse gut microbiota and a high-fiber and antioxidant-rich and plant-based diet reduce inflammation, whereas regular physical activity, which acts as a key protective factor of digestive health, supports gastrointestinal motility. Psychological well-being plays an important role in maintaining digestive health. Certain genetic traits, including longevity-related gene variants, also help protect against age-related declines in digestive function. Centenarians often exhibit distinct physiological characteristics that support sustained digestive health. Gaining insight into these mechanisms can guide the development of strategies to promote healthy aging and enhance quality of life in older adults, contributing to a longer and healthier lifespan.
